# How does technology pathway choice influence economic viability and environmental impacts of lignocellulosic biorefineries?

**DOI:** 10.1186/s13068-017-0959-x

**Published:** 2017-11-14

**Authors:** Karthik Rajendran, Ganti S. Murthy

**Affiliations:** 1 Department of Biological and Ecological Engineering, Oregon State University, Corvallis, OR 97331 USA; 2 MaREI Centre, Environmental Research Institute, University College Cork, Cork, Ireland; 3 School of Engineering, University College Cork, Cork, Ireland

**Keywords:** Techno-economic analysis, Life cycle assessments, Lignocelluloses, Biorefinery, Biomass pretreatment, Process simulation, Systems analysis, Advanced biofuels

## Abstract

**Background:**

The need for liquid fuels in the transportation sector is increasing, and it is essential to develop industrially sustainable processes that simultaneously address the tri-fold sustainability metrics of technological feasibility, economic viability, and environmental impacts. Biorefineries based on lignocellulosic feedstocks could yield high-value products such as ethyl acetate, dodecane, ethylene, and hexane. This work focuses on assessing biochemical and biomass to electricity platforms for conversion of Banagrass and Energycane into valuable fuels and chemicals using the tri-fold sustainability metrics.

**Results:**

The production cost of various products produced from Banagrass was $1.19/kg ethanol, $1.00/kg ethyl acetate, $3.01/kg dodecane (jet fuel equivalent), $2.34/kg ethylene and $0.32/kW-h electricity. The production cost of different products using Energycane as a feedstock was $1.31/kg ethanol, $1.11/kg ethyl acetate, $3.35/kg dodecane, and $2.62/kg ethylene. The sensitivity analysis revealed that the price of the main product, feedstock cost and cost of ethanol affected the profitability the overall process. Banagrass yielded 11% higher ethanol compared to Energycane, which could be attributed to the differences in the composition of these lignocellulosic biomass sources. Acidification potential was highest when ethylene was produced at the rate of 2.56 × 10^−2^ and 1.71 × 10^−2^ kg SO_2_ eq. for Banagrass and Energycane, respectively. Ethanol production from Banagrass and Energycane resulted in a global warming potential of − 12.3 and − 40.0 g CO_2_ eq./kg ethanol.

**Conclusions:**

Utilizing hexoses and pentoses from Banagrass to produce ethyl acetate was the most economical scenario with a payback period of 11.2 years and an ROI of 8.93%, respectively. Electricity production was the most unprofitable scenario with an ROI of − 29.6% using Banagrass/Energycane as a feedstock that could be attributed to high feedstock moisture content. Producing ethylene or dodecane from either of the feedstocks was not economical. The moisture content and composition of biomasses affected overall economics of the various pathways studied. Producing ethanol and ethyl acetate from Energycane had a global warming potential of − 3.01 kg CO_2_ eq./kg ethyl acetate.

**Electronic supplementary material:**

The online version of this article (10.1186/s13068-017-0959-x) contains supplementary material, which is available to authorized users.

## Background

The world liquid fuels consumption as of Q1 2017 was 97 million barrels per day and it is expected to reach 100 million barrels per day in the next 2 years [1]. In 2012, the jet fuel consumption was 5.3 million barrels per day [1]. For January 2017, 63.5 million tons of coal were used to partially meet the electricity demand in the USA [1]. The transportation sector consumes 25% of the overall energy consumed and it is expected to rise at the rate of 1.4% every year [2].

**Fig. 1:**
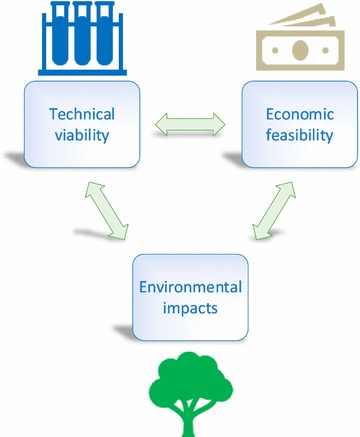
Factors in sustainability affecting an industrial process

The term industrial sustainability is based on technical feasibility, economic viability and environmental sustainability of a product or process [3] (Fig. 1). Several attempts have been made in the past to improve the commercialization potential of lignocellulosic ethanol using various approaches such as increasing enzyme efficiency, novel pretreatment methods [4, 5], and high solids loading of biomass [6–9]. However, the production cost of ethanol produced from lignocellulosic biomass is still not decisively lower than that of gasoline and hence commercialization of lignocellulosic ethanol is still very limited. Additionally, the recent drop in crude oil prices has adversely affected the outlook for lignocellulosic ethanol production.

Several techno-economic analyses and life cycle assessment (LCA) studies had been carried out for the lignocellulosic ethanol production to quantify the economic viability and environmental impacts. Techno-economic studies have focused on various aspects including the effect of pretreatments on ethanol production, integrating first and second generation ethanol production [10], using different feedstocks, and effect of an increase in enzyme yields [11–18]. For the LCA, most of the studies had focused on feedstocks, comparing ethanol production processes with other value-added products, and the impact of various allocation methods on the LCA results [19–26].

The concept of lignocellulosic biorefinery has been defined analogously to petroleum refinery as a processing facility/strategy that produces multiple products such as polymers, composites, biochemicals, specialty chemicals, polyols, and energy in addition to liquid biofuels [27]. In petroleum, refinery petrochemicals contribute a larger fraction of revenues on a normalized mass basis compared to energy products. Similarly, the rationale for biorefinery is to produce high-value product streams that could match or replace the products produced from the petrochemical route while simultaneously producing fuels. It is essential to perform an integrated techno-economic and environmental impact analysis to understand the overall impact of producing various products from lignocelluloses [28]. Some reports have analyzed the above-mentioned three-dimensional factors of industrial sustainability for pyrolysis of lignocellulosic biomass, ethanol and butanol production [29–33]. Very few studies had considered the comprehensive integrated analyses with techno-economic analysis and LCA of the biochemical and biomass to electricity platforms with a focus on product pathways from lignocelluloses such as [34] which looked at revitalizing sugar industry through multi-product biorefineries from lignocelluloses. This work widens the scope to include additional feedstocks and focuses on biochemical/bioproducts such as ethyl acetate, ethylene and dodecane where the other work was focused on butanol, ethanol, acetone, methanol and furfural. This shows that the biobased products are plentiful and need further exploration.

As noted above, there are many literature studies describing the technologies for conversion of biomass into biofuels and bioproducts. However, it is imperative to understand the implications of the technology pathways choice on the economic viability and environmental sustainability to develop technologies that are commercially successful and have low environmental impacts. The overall goal of this paper is to develop a framework to conduct such analyses to assess the implications of technology pathways on the economic viability and environmental impact metrics.

The objective of this work is to perform techno-economic analysis (TEA) and life cycle assessment (LCA) using two lignocellulosic feedstocks, i.e., Banagrass and Energycane in a biorefinery that produces products such as electricity, ethanol, ethyl acetate, ethylene, dodecane, and hexane. Six process models were developed using Intelligen Superpro Designer^®^ for each biomass including (1) Dilute sulfuric acid pretreatment for ethanol production (BE, EE representing Banagrass-to-Ethanol and Energycane-to-Ethanol pathways, respectively), (2) Ethanol (from hexoses) and ethyl acetate (conversion of pentoses to acetic acid followed by reactive distillation with ethanol) production (BEEA, EEEA), (3) Ethyl acetate production by converting both hexoses and pentoses to acetic acid and using ethanol from external sources for reactive distillation (BEA, EEA), (4) Ethylene production by dehydration of ethanol (BET, EET), (5) Dodecane or jet fuel production from ethylene (BD, ED), and (6) Biomass to electricity (BEL, EEL). Techno-economic assessments were performed to assess technical viability and economic feasibility, while life cycle assessments were performed to assess the environmental impacts of various biorefinery technology pathways. The plant size in this study was set at 60,000 dry MT/year of biomass. Sensitivity analysis was carried out for the crucial factors such as the cost of the main product, enzymes, biomass and different capacities. Dil. acid pretreatment for ethanol production (BE and EE) was considered as the base scenario for other processes. The process models and other supplementary information are provided to promote transparency and facilitate replication of our results.

## Results and discussion

**Fig. 2:**
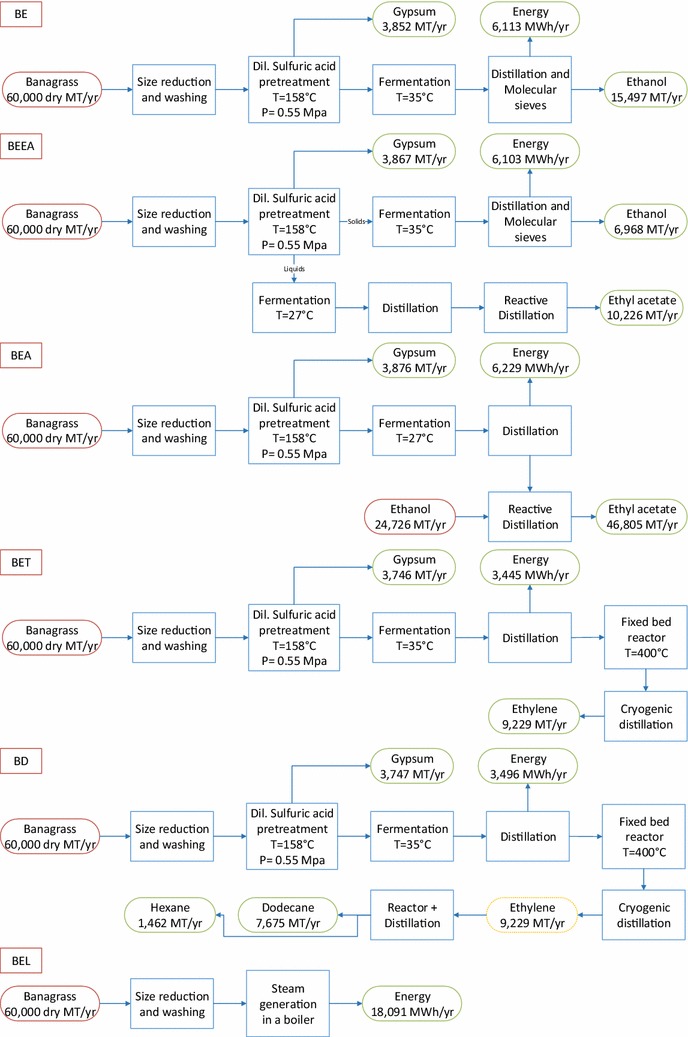
Schematics of different processes using Banagrass as a feedstock

**Fig. 3:**
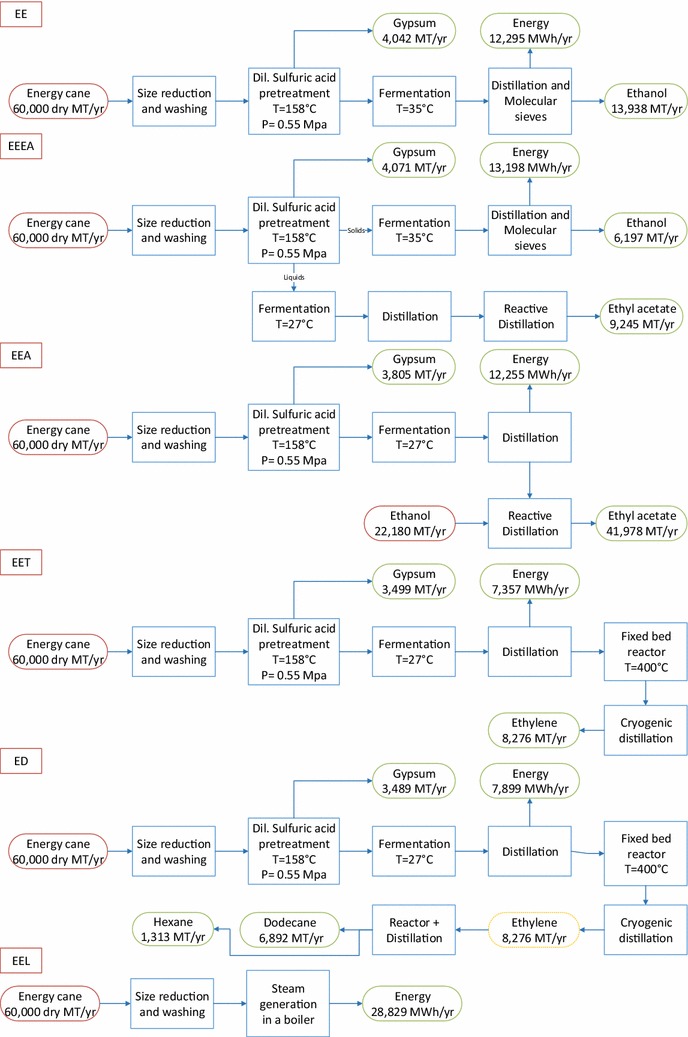
Schematics of different processes using Energycane as a feedstock

The process simulation for the different products was performed using multiple pathways including the production of biofuels, biochemicals, advanced biofuels and electricity. The overall process schematic and the overall mass balance for all the scenarios is shown in Figs. 2 and 3 for Banagrass and Energycane, respectively. All the simulation files are presented in the Additional files 1-12 to increase the transparency and facilitate reproduction of the results.

### Technical feasibility

Two feedstocks, Banagrass and Energycane were considered for producing bioproducts including ethanol, ethyl acetate, ethylene, dodecane, hexane, and electricity. Banagrass feedstock processing pathways, in general, had higher product yields compared to Energycane, which could be attributed to a higher fraction of fermentable sugars (Table 1). For example, ethanol production in scenario BE was 11% higher than EE scenario. In contrast, the electricity production for BEL was 37% lower than electricity produced in EEL scenario. Lower electricity production for BEL was mainly attributed to the lower lignin and extractives fraction and higher moisture content in Banagrass compared to Energycane, which result in lower LHV values compared to Energycane. The consumption of different raw materials was directly proportional to the composition of the biomass (Table 2). The annual enzymes requirement in Energycane was 11% lower compared to Banagrass scenario due to the higher cellulose content (37.48% in Banagrass vs. 33.44% dry basis in Energycane) in Banagrass. Table 3 summarizes the utility consumption for different scenarios. In all the scenarios, both high-pressure (HP) steam (250 °C; 4.0 MPa) and low-pressure (LP) steam (152 °C; 0.4 MPa) were produced in-house from the boiler by combusting lignin and was sufficient to meet/exceed the plant requirements for these utilities. The HP steam was primarily used in the pretreatment reactor and other operations required the use of LP steam. Similarly, all the electricity needs of the plant were met/exceeded by the electricity production in the plant.

**Table 1:** The composition of biomass used in this study

Composition	Banagrass		Energy cane	
	Wet basis (%)	Dry basis (%)	Wet basis (%)	Dry basis (%)
Cellulose	10.22	37.48	10.05	33.44
Hemicellulose	6.39	23.43	6.36	21.16
Lignin	4.49	16.46	3.78	12.58
Extractives	3.56	13.05	7.92	26.36
Ash	2.61	9.57	1.94	6.46
Moisture	72.73	–	69.95	–

**Table 2:** List of raw materials used in different scenarios for this study

Banagrass	Unit (MT)/year					
	BE	BEEA	BEA	BET	BD	BEL
Water	34,618	39,327	67,777	107,234	102,112	
Gasoline	147	70				
Calcium hydroxide	1762	1769	1773	1711	1712	
Sulfuric acid	2571	3071	3531	2498	2498	
Banagrass (dry MT)	60,000	60,000	60,000	60,000	60,000	60,000
Cellulase	4259	4377	4259	4259	4259	
DAP	20	20	8	20	20	
Yeast	50	50		50	50	
Ethanol			24,726			
Nitrogen				377	377	
Diborane (kg)					2	
Nickel (kg)					25	

Energy cane	Unit (MT)/year					
	EE	EEEA	EEA	EET	ED	EEL
Water	41,291	45,907	99,413	134,564	106,626	
Gasoline	139	62				
Calcium hydroxide	1862	1877	1750	1604	1600	
Sulfuric acid	2718	3225	3403	2341	2335	
Energy cane (dry MT)	60,000	60,000	60,000	60,000	60,000	60,000
Cellulase	3800	3930	3800	3800	3800	
DAP	20	20	8	20	20	
Yeast	50	50		50	50	
Ethanol			22,180			
Nitrogen				339	339	
Diborane (kg)					2	
Nickel (kg)					25	

All the values mentioned above were rounded to the nearest metric tons

**Table 3:** Different annual utility consumption summary for different scenarios

	BE	BEEA	BEA	BET	BD	BEL
Banagrass						
Power consumption (kW-h)	(6,491,000)	(7,473,000)	(7,079,000)	(9,433,000)	(9,483,000)	(4,472,000)
Power production (kW-h)	12,604,000	13,576,000	13,308,000	12,878,000	12,979,000	22,563,000
Steam (MT)	(147,000)	(183,000)	(385,000)	(140,000)	(141,000)	
Cooling water (MT)	9,396,000	9,535,000	13,871,000	8,255,000	8,346,000	
Chilled water (MT)	12,000	1,358,000	4,515,000	1,007,000	1,007,000	
CT water (MT)	6,637,000	11,647,000	6,206,000	6,469,000	6,469,000	
Steam high P (MT)	(26,000)	(26,000)	(26,000)	(25,000)	(25,000)	
Cryogenic cool (MT)				91,000	91,000	
Hot water (MT)					9000	

	EE	EEEA	EEA	EET	ED	EEL
Energycane						
Power consumption (kW-h)	(5,684,000)	(5,831,000)	(6,756,000)	(10,918,000)	(10,496,000)	(4,090,000)
Power production (KW-h)	17,979,000	19,029,000	19,011,000	18,275,000	18,395,000	32,919,000
Steam (MT)	(153,000)	(188,000)	(384,000)	(133,000)	(135,000)	
Cooling water (MT)	9,231,000	9,739,000	13,892,000	7,777,000	8,312,000	
Chilled water (MT)	11,000	1,418,000	4,527,000	933,000	934,000	
CT water (MT)	6,721,000	11,804,000	6,399,000	6,504,000	6,500,000	
Steam high P (MT)	(27,000)	(27,000)	(25,000)	(23,000)	(23,000)	
Cryogenic cool (MT)				81,000	82,000	
Hot water (MT)					8000	

The values within the parenthesis indicates that the utility was produced onsite, and hence a negative value was mentioned

The ethanol yield of 295.8 L/dry MT using Energycane in this study (EE) were comparable to the NREL reported yields of 298.62 L/dry MT for corn stover [35]. Similar results (252.62 L/dry MT) were reported by Kumar et al. using grass straw as a feedstock [36]. Contrasting to Energycane and other reported literature, Banagrass yielded 392 L/dry MT ethanol. GREET reported around 58.8% conversion efficiency of converting ethanol to ethylene, while this study estimated this conversion efficiency as 59.5% (Figs. 2; 3 BET and EET) [37].

Electricity was produced as a coproduct in all scenarios where Banagrass yielded less electricity overall, in comparison with Energycane. This is mainly due to the reason that Banagrass had high cellulose and hemicelluloses, which were converted to various products depending on the pathway resulting in higher electricity consumption during the production processes. Additionally, the amount of residues from the processes using Banagrass as a feedstock was lower as major fractions such as cellulose and hemicellulose are already utilized resulting in less feedstock for boilers resulting in lower electricity production. For biochemical process using Banagrass as a feedstock, the electricity production as a coproduct was in the range of ca. 3500 and 6200 MWh/year. Scenarios including BE, BEEA, BEA produced more than 6000 MWh while lower amounts were produced in BET and BD scenarios, due to the large amount of electricity utilized in ethylene production. Similar trends were seen for the Energycane as well where electricity production was in the range of ca. 7300 and 13,200 MWh/year.

### Economic viability

**Fig. 4:**
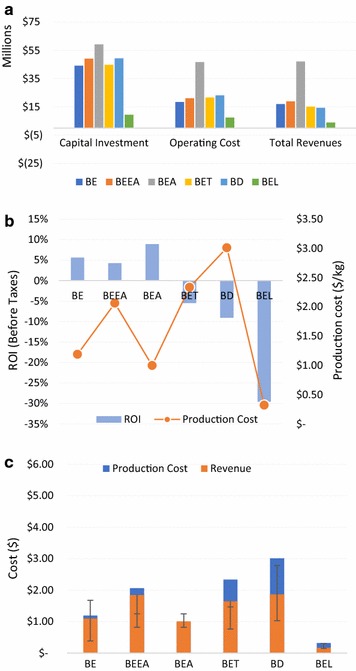
Economic indexes for different scenarios using Banagrass as a feedstock. Capital cost, operational cost and revenues (**a**). Return on investment and production cost (**b**). Production cost, production revenue and the error bar indicates the historical prices in the last decade (**c**)

**Fig. 5:**
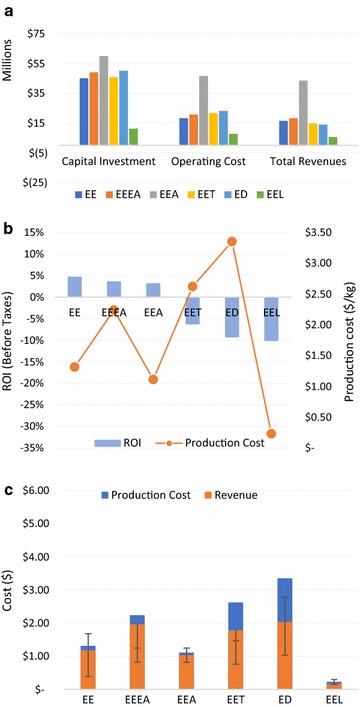
Different economic indexes for different scenarios using Energycane as a feedstock. Capital cost, operational cost and revenues (**a**). Return on investment and production cost (**b**). Production cost, production revenue and the error bar indicates the historical prices in the last decade (**c**)

Economic analysis was used to quantify some of the important economic indicators such as capital investments, operational investments, revenue, production cost, production revenue, and return on investments (Figs. 4 and 5). The capital investment for the biochemical product pathways varied between $44 and $59 Million. The OPEX to CAPEX ratio for biochemical product pathways except BEA and EEA was 0.42–0.49 and was 0.78 and 0.79 for EEA and BEA, respectively. This increase could be attributed to an increase in the raw materials costs, as ethanol was purchased in bulk from outside sources (assumed to be corn ethanol) to produce ethyl acetate in the EEA and BEA scenarios. Compared to biochemical ethanol production, the thermochemical processes for producing electricity had lower capital investment. The CAPEX for electricity production was $9.5 and $11.2 Million for BEL and EEL scenarios, respectively.

The production cost of ethanol from the Banagrass and Energycane was $0.93 and $1.03/L, respectively, and is comparable to reported values of $0.7–1.2/L lignocellulosic ethanol in literature [12–14, 36, 38]. The production cost of ethyl acetate was lower when ethanol was purchased from a corn ethanol plant (BEA—$1.00/kg and EEA—$1.11/kg) than it was produced on site from hexoses (BEEA—$2.06/kg and EEEA—$2.24/kg). The difference between the production cost for BEEA and BEA was $1.06/kg, while for EEEA and EEA it was $1.13/kg. It was evident from the results that on-site ethanol production (BEEA or EEEA) in comparison with ethanol purchased elsewhere (BEA or EEA) for ethyl acetate production process was expensive due to three reasons: (1) The selling cost of ethyl acetate was 10¢/kg higher than ethanol (In scenario BEEA or EEEA a fraction was sold as ethanol), (2) The amount of ethyl acetate produced was higher in comparison with ethanol, (3) The carbon efficiency between the acetic acid productions vs. ethanol fermentation, where about 50% carbon is lost in the form of carbon dioxide during hexose fermentation to ethanol.

The production cost of dodecane and ethylene was expensive compared to all other scenarios, and it was primarily attributed to the high electricity consumption for the ethylene production. The loss of revenue from electricity for scenarios BET, EET, BD, and ED increased the production cost, which resulted in these scenarios being not profitable. The production costs of electricity using thermochemical processes were $0.32 and $0.23/kW-h for BEL and EEL, respectively. From an economical point of view, it was apparent that producing electricity was not profitable due to its high moisture content. This indicates the tradeoffs associated with processing green biomass that has not been field dried. Due to high moisture content, green biomass with high moisture content is more suitable for biochemical processing than thermochemical processing due to its low LHV.

The ROI was highest for scenarios BEA and BE, i.e., 8.9 and 5.6%, respectively. The ROI for scenarios BD and ED was − 9.0 and − 9.3% correspondingly. Most of the scenarios showed a trend of lower production revenues than its production cost, which could be attributed to the current market prices of various products due to depressed crude oil prices. The difference between production cost, revenues, and the historical prices in the last decade are presented in Figs. 4c and 5c. Given the dynamics of market fluctuations on the prices, scenarios including BE, EE, BEL, EEA, and EEL would make profits if the crude oil prices rise above $80/barrel. A graph was plotted between the historical market prices between 2007 and 2016 for ethanol, jet fuel and ethylene vs. crude oil, to establish a relationship between crude oil and the profitability of bio-based products (Additional file 13).

### Sensitivity analysis

**Fig. 6:**
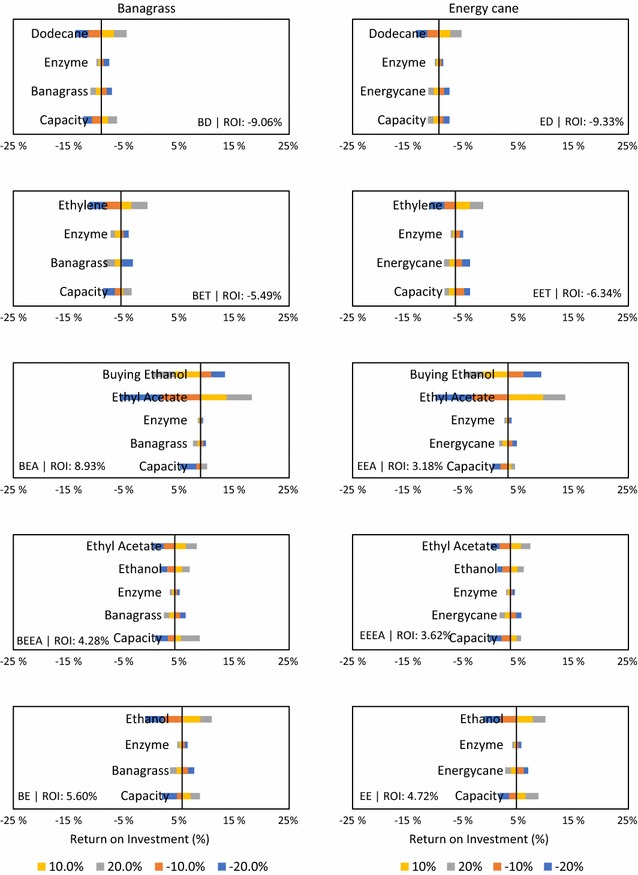
Sensitivity analysis of important parameters for the different scenarios considered in this study

A comprehensive sensitivity analysis for all the twelve scenarios considering some of the important parameters was performed to assess the importance and influence of the important parameters values on the reliability of the techno-economic analysis. The economic indicator used in this study for the sensitivity analysis was ROI. The important parameters include the biomass cost, enzyme cost, main product cost, ethanol cost and the plant capacity (Fig. 6). Ethanol selling price was the most sensitive parameter for BE and EE scenarios where selling ethanol at 20% higher resulted in ROI increase to 11.0 and 9.96%, respectively, from the base case result of 5.6 and 4.72%. The most profitable scenario (BEA) in the base case was affected by two important parameters, i.e., the selling price of ethyl acetate and purchasing cost of ethanol. When the price of ethyl acetate was altered by ± 20%, ROI was altered between − 5.7 and 18.2% compared to the base case value of 8.93% ROI. For ethylene and dodecane scenarios, increasing the selling price of the product to + 20% did not improve the ROI. For + 20% increase in selling price, BD and ED yielded − 4.47 and − 5.25%, respectively. This shows that advanced biofuels production or jet fuels using lignocelluloses will not be an economically viable option unless there are significant cost increases in the crude oil prices (Additional file 13).

The sensitivity analysis based on the composition and moisture content revealed that a decrease in moisture content increased the ROI in most of the scenarios. The fluctuation in the composition did not affect the ROI with a difference of ± 1% (Additional file 14). Compared to the variation in composition, the moisture content of the feedstock at plant gate had a greater effect on profitability. This highlights the tradeoffs associated with wet harvesting and field-dried biomass. While field-dried biomass can be transported and stored relatively easily compared to wet biomass, energy requirements for size reduction of dry biomass are relatively higher compared to wet biomass. Additionally, since the wet biomass is processed as soon as it arrives in the plant, the requirements for storage are greatly minimized, and the water requirements in the plant can be partly/completely met with the water in the wet feedstock. In general, low moisture biomass showed high profitability (data not shown).

An analysis of the results for all scenarios indicated that the results are influenced by relatively few key factors. Some of the factors such as the biomass cost, selling price of the product and the capacity of the plant were key determinants of the economic feasibility of various technology pathways. It is worth noting that producing high-value products is not always economically feasible unless the choice of pathway considered was simple. This is reflected in the analysis where the biomass to electricity pathway had the highest profitability when compared to biochemical pathways. It is essential that the values for the key drivers be determined accurately when considering scale-up scenarios. The cost of biomass used in this study was $80/dry MT and reducing the cost could change the dynamics of the profitability. External factors such as the product price affect the viability of the technology pathways. The price of the crude oil largely determines the prices of the products investigated in this study, and thus the future economic feasibility of biorefineries producing these products will be largely dependent on crude oil prices in the absence of any government support for biofuels (Additional file 13).

### Environmental impacts

#### Carbon balance

**Fig. 7:**
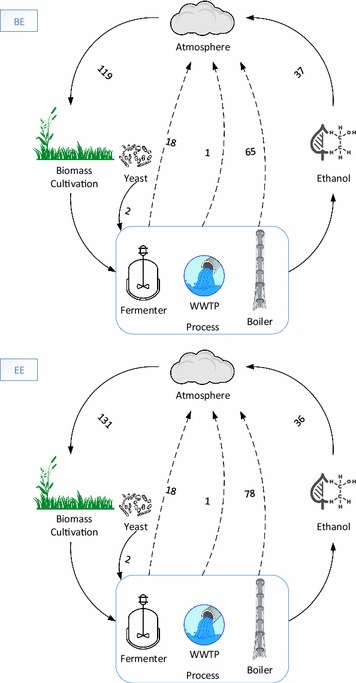
Elemental carbon balance in kilogram for every ton of biomass (wet basis) produced in BE and EE scenario

Carbon balance was performed to increase the transparency of the analysis and provide information on the carbon flows through the various processes (Fig. 7). Cultivation of biomass results in 119 and 131 kg carbon sequestration in the form of carbon dioxide for Banagrass and Energycane, respectively. This sequestered carbon was modified to different forms of carbon during the process to produce various products/fuels/chemicals. Within the process flows, the carbon balance could be partitioned into four forms: carbon release in the form of carbon dioxide from fermentation, wastewater treatment, and boiler for steam generation from lignin and carbon content in the product, i.e., ethanol. The carbon was released from the process in two forms: (1) Ethanol and (2) Carbon dioxide. The emission when ethanol runs as a fuel in an engine will convert ethanol to carbon dioxide, which returns to the atmosphere completing the carbon cycle.

Of the amount of carbon sequestered, 15% (BE) and 14% (EE) was discharged back to the atmosphere from the fermentation process, where the microbes release carbon dioxide. About 55 and 59% sequestered carbon was released during combustion in the steam boiler in the BE and EE processes, respectively. The other major carbon release was from the carbon in the ethanol where 31–27% of sequestered carbon was present and would be released during the fuel combustion. It is also important to note that some minor carbon flows were added during the process in the form of yeast.

#### Life cycle assessments

**Fig. 8:**
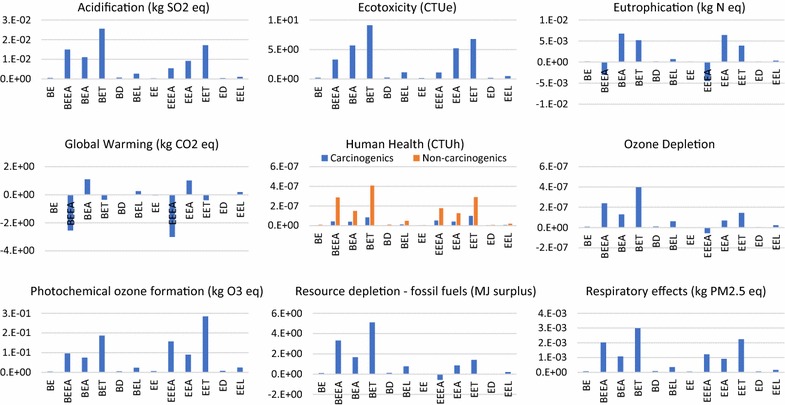
Environmental impacts for different scenarios using TRACI 2.1 as impact assessment method. The functional unit for different scenarios was mentioned based on their mass or energy. Ethanol, dodecane—MJ, ethyl acetate, ethylene—kg, electricity—kWh

Based on the inputs from TEA, LCI inputs were obtained. LCA was assessed with LCI using OpenLCA and TRACI 2.1 was used as an impact assessment method (Additional file 15). Ten different indicators were compared between the twelve scenarios of which seven were environment related and the other three were human health related (Fig. 8). The functional units for energy-based products such as ethanol, dodecane, and electricity had an energy-based functional unit MJ, whereas chemicals such as ethyl acetate, ethylene had a mass-based functional unit (kg). The allocation was avoided using the system expansion method where other coproducts replaced equivalent products and indicated as avoided products. This is the preferred method as per the ISO 14044 (2006) standards for LCA [39]. Acidification relates to the increasing concentration of hydrogen ion which leads to the addition of acids into the environment [40] and was measured in terms of kg SO_2_ equivalent. Acidification was highest for the scenario BET with 25.5 g SO_2_ eq. Acidification was lowest when ethanol was the only product produced (5.4 × 10^−4^—BE, EE—3.1 × 10^−4^ kg SO_2_ eq.), and any additional process modifications to diversify the product mix increases the acidification to the environment.

Eutrophication relates to the augmentation of the aquatic ecosystem with nutrients which increases the growth of algae and other weeds [41] and was measured in terms of kg N Eq. Eutrophication for scenario BE and EE was 1.1 × 10^−4^ and 7.17 × 10^−5^ kg N Eq., respectively. Compared with other scenarios only BEEA and EEEA wherein plant ethanol and ethyl acetate were produced had a negative eutrophication, which means that these scenarios reduce the eutrophication effects when produced due to the replacement of the fossil fuel-based products. BEEA and EEEA had an eutrophication metric of − 3.07 × 10^−3^ and − 4.51 × 10^−3^ kg N Eq., respectively. The jet fuel equivalent scenarios BD and ED had an eutrophication between 1.3 × 10^−4^ and 9.6 × 10^−5^ kg N Equivalent.

Ozone depletion refers to the exhaustion of ozone layer which safeguards from radiation. If ozone layer is damaged it could lead to skin cancers and cataracts and in addition, it also affects crop cultivation, plants and marine life [42]. Substances which deplete ozone layer were chlorofluorocarbons (CFC) which were used in refrigerants, solvents and foaming agents. Hence, ozone depletion was measured in kg CFC-11 Eq. Apart from EEEA, the rest of the scenarios had a positive ozone depletion. The ozone depletion was highest for the scenario BET 3.97 × 10^−7^ kg CFC-11 Equivalents.

Resource depletion corresponds to the reduction of fossil fuels [40, 41] and was quantified with respect to fossil fuels as MJ surplus in TRACI 2.1. Scenarios such as BET, BEEA, BEA, and EET had a highest positive resource depletion in the order of 5.1, 3.31, 1.68, 1.41 MJ surplus for every kg of the main product produced. Human health was represented in the form of respiratory effects, carcinogenics, and non-carcinogenics. Respiratory effects were measured as particulate matter kg PM2.5 Eq. whereas the other two health effects were measured as CTUh (Comparative Toxic Unit for Human) [40, 42]. In general, non-carcinogenics had higher impacts compared with carcinogenics. Scenarios BET and EET had the highest non-carcinogenic emission 4.07 × 10^−7^ 2.91 × 10^−7^ CTUh, respectively. Similarly, respiratory effects were also highest for the scenarios BET, EET, BEEA, and EEEA.

Global warming refers to the average increase in the temperature of the atmosphere, which could be caused by both natural and human-induced processes [40–42]. Global warming potential is measured in terms of kg CO_2_ eq. in TRACI 2.1 Ethanol producing scenarios BE and EE had a global warming of − 12.3 and − 40 g CO_2_/MJ ethanol, respectively. Most of the scenarios had a negative global warming except where ethanol was bought from elsewhere (BEA and EEA) and thermochemical processes (BEL and EEL).

Similar to the economic feasibility results, the LCA results were also determined by few key factors. For example, displaced electricity played a critical role in results that indicated lower environmental impacts of biofuels in Hawaii. In Hawaii, most of the electricity was produced from diesel and coal that had relatively higher GHG emissions and displacing them with bio-based electricity resulted in lower environmental impacts (primarily lower GHG emissions) for various biochemical and biomass to electricity technology pathways. Thus the advantages of the biorefineries were more pronounced compared to the mainland US. The other driver that affected the LCA result was the impacts associated with the petrochemical products which were displaced by bio-based sources.

### Comparison of data with literature

**Fig. 9:**
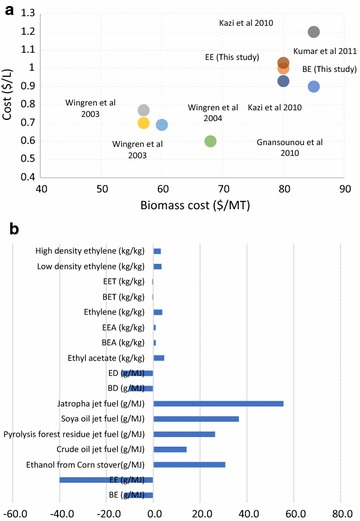
Comparison of cost estimates for ethanol production with literature reported values (**a**) and GHG emission estimates for different fuels and chemicals with respect to values reported in GREET (**b**). For GHG emissions, a respective functional unit for each category is mentioned

The production cost estimates of ethanol from this study were compared with the previously reported literature [12–14, 36, 38]. The production cost of ethanol in this study for BE and EE were $0.93/L and $1.03/L, respectively. Figure 9 shows the data comparison for production cost estimates versus the biomass cost. The other study reported had a production cost estimates varying between $0.6 and $1.2/L [12–14, 36, 38]. The ethanol production cost was higher compared to other studies and is primarily attributable to the smaller size of the plant in this study (60,000 dry MT/year) compared to a four-time higher operational capacity in other studies which results in lower ethanol production costs. An additional reason was the cost of biomass which has a direct effect on the cost of production ($80/dry ton was used in this study). A two-decade-old study reported the production cost of ethylene at $0.55/kg (inflation adjusted), while this study reported $2.34/kg, which was four times higher [43]. Similarly, a study by Pearlson et al. reported the production of jet fuel from vegetable and animal fats by extracting esters and fatty acids reported a production cost of $1.16/L, whereas this study reported the production cost estimate between $2.25 and 2.51/L [44]. The feedstock considered in the Pearlson et al. study was a lipid source with lower processing costs, whereas this study considered the depolymerization and polymerization which had increased the production cost of jet fuels in addition to the moisture content of the substrate.

The environmental impacts of the global warming reported in this study for the ethanol production were − 12.3 and − 40 g CO_2_ eq./MJ ethanol. GREET [37] reported a WTP global warming of 30.8 g CO_2_ eq./MJ from corn stover ethanol, whereas Luo et al. reported 50 g CO_2_ eq./MJ ethanol produced [45]. Cavalett et al. reported the global warming potential for the lignocellulosic ethanol of 24.2 g CO_2_ eq./MJ ethanol [46]. The results of this study considered displacing the electricity production in Hawaii which is mainly based on fossil fuels and therefore had a stronger impact on lowering global warming potential. Similarly, for ethyl acetate production from renewable sources by Thuy et al. reported 1.0 kg CO_2_ eq./kg of ethyl acetate produced, whereas this study estimates a global warming potential of 1.1 and 1.02 kg CO_2_ eq./kg [47]. For jet fuel production, Shonnard et al. reported the global warming potential of 25–30 g CO_2_/MJ jet fuel from camelina, and this study reported between − 10.2 and − 13.3 g CO_2_/MJ jet fuel [48]. In general, the results from both techno-economic and environmental impacts analysis agreed with the literature reported earlier. Figure 11 shows the comparison of different global warming potential for different fuels and chemicals from GREET and this study.

## Conclusion

Six biorefinery technology pathways including biochemical and thermochemical (electricity from biomass) methods using two lignocellulosic feedstocks (Banagrass and Energycane) were evaluated for technical feasibility, economic viability, and the environmental impacts. The process models developed were designed based on 60,000 dry MT/year biomass processing facility. The capital investment ranged between $40 and 60 Million for the liquid fuel/chemical production scenarios, while the BEL and EEL had a capital investment between $9 and 11 Million. Techno-economic analysis revealed that scenario BEA was the most economical overall (ethyl acetate) with a payback period of 11.2 years, while electricity production (BEL and EEL) was the  most unprofitable due to high moisture content in the biomass. Producing advanced biofuels (dodecane) was not economically feasible and the difference in their unit production cost and unit production revenue was − $1.14/kg dodecane (BD). An increase in prices of the products aided by increased crude oil prices could make some of these scenarios economically viable. Environmental impact analysis revealed that scenario BEEA and EEEA scenarios result in lower environmental impacts such as reduced global warming potential. Producing ethyl acetate from Banagrass was the most beneficial scenarios in terms of technical, environmental and economic metrics.

## Methods

### Biomass

In this study, Banagrass and Energycane (Table 1) were considered as two biomass sources. The composition of Banagrass was obtained from compositional analysis using standard NREL protocols [49], while Energycane composition was based on results reported by Kim and Dale [50]. The harvest data for both the biomasses were obtained from an experiment field trials conducted on Hawaii island. The biomasses were harvested at the rate of 15 MT/acre which had a harvest efficiency of 90%. The capacity considered in this study was based on 60,000 dry MT biomass/year.

### Assumptions and justifications

The site for the proposed plant was on Maui Island, Hawaii where diesel-based electricity is the primary source of electricity in stark contrast to the mainland USA scenario. It is expected that global warming potential and other environmental impacts would be different from the mainland USA due to the different fuel sources for electricity production in Hawaii and Mainland USA. The choice of plant size was 60,000 dry MT/year was based on the land availability in Maui considering the important aspects including crop yield and crop rotation. While the plant size is small considering the mainland USA, this was the most feasible capacity for this particular location. Feedstock including Banagrass and Energycane was chosen as several pilot field trials were conducted in Maui on crop yield, emissions, nutrient requirements, water, electricity, and agricultural machinery inputs. These data were used in life cycle inventory and assessments.

### Model development

**Fig. 10:**
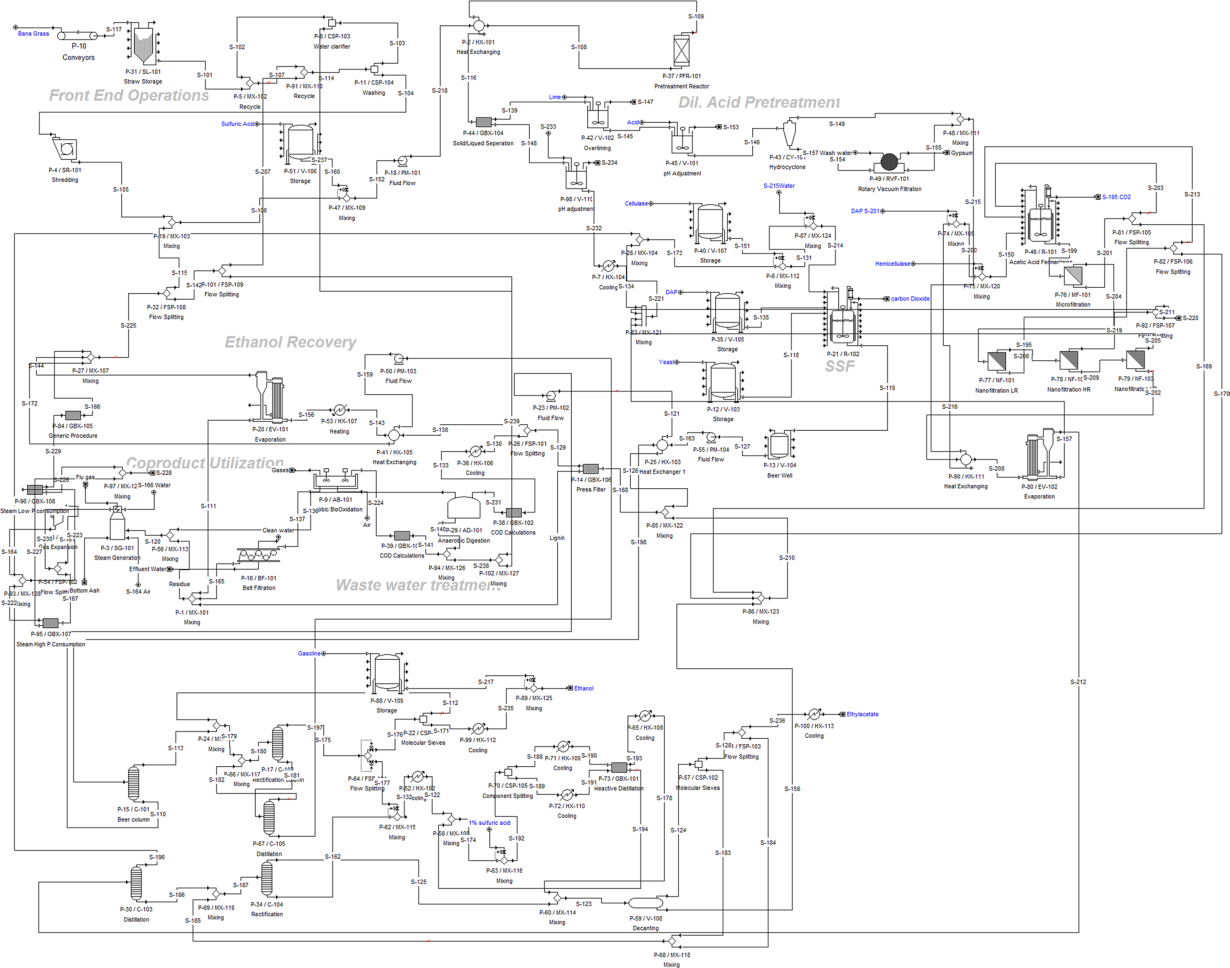
Snapshot of the process flowsheet developed in Superpro designer for the case BEEA

For the process simulations, 12 scenarios were developed including six for each feedstock. The six scenarios were coded based on their feedstock and the main product produced in that scenario. For example, Energycane was coded as “E”, ethylene was coded as “ET”, and this scenario was called as EET. Similarly, the scenario producing electricity using Banagrass was coded as BEL. For the base case, 12 simulations were performed which included around 100 unit operations in each flowsheet. Detailed process models for BEEA scenario BEEA highlighting the different sections in that process is shown in Fig. 10.

#### Dil. acid pretreatment for ethanol production (BE and EE)

Banagrass and Energycane were transported to the site, where the field-dried biomass is conveyed to a silo for 10 days before further processing. The stored biomass was washed to remove any trash, dirt or debris before size reduction in a knife mill. About 0.75% (w/w) sulfuric acid was added to the incoming biomass and sent to the pretreatment reactor operating at 158 °C and 0.55 Mpa. The pretreated lignocelluloses were centrifuged to separate into solid and liquid fractions. The solid fractions predominantly contain celluloses, whereas the liquid fraction contains dissolved solids and hemicelluloses. The liquid fraction was overlimed and the pH was adjusted, where calcium sulfate was precipitated as gypsum (byproduct) through hydro-cyclone and purified through vacuum filtration.

The cellulose-rich solid stream after solid/liquid separation was pH adjusted and cellulase enzymes were loaded at the rate of 20 mg/g cellulose [35]. Along with yeast and diammonium phosphate, simultaneous saccharification and fermentation (SSF) were performed with a retention time of 5 days [35]. About 5% (W/W) ethanol was obtained after SSF process, which was distilled in three columns. The first column was a beer column, where ethanol was concentrated to about 38–40% (W/W), followed by a rectification column where ethanol was further concentrated to 42–45% (W/W). After the third rectification column, the ethanol concentration was ~ 93% (W/W) which was further purified to 99.5% using molecular sieves, to break the ethanol–water azeotrope. The purified ethanol is cooled and denatured with 1% gasoline before it is sold as the main product.

The bottom stream from the beer column containing lignin along with other liquid fraction was press filtered to recover lignin as a solid fraction, which was combusted in the boiler for steam generation and electricity production. The liquid fraction was split to recover leftover ethanol in multi-effect evaporator, whereas 25% of the liquid stream was sent for waste water treatment (WWTP). The WWTP includes anaerobic digestion, aerobic oxidation, and belt filtration. In the anaerobic digestion process, methane was recovered and directed to the boiler along with lignin and sludge (after WWTP). In the boiler, the air was pumped at the rate of 10% oxygen in excess to produce steam at 257 °C and 4.5 MPa [51, 52]. The flue gas exit temperature was set at 200 °C, and elemental composition of the individual component in the boiler was mentioned. The high-pressure steam was used in the gas turbine for gas expansion and electricity production. Part of the high-pressure steam was also used for meeting the heating requirements in the pretreatment reactor. The water from boiler, evaporator, and rectification column was recycled back to the process as modeled in NREL model [35] and Kumar et al. [36]. All the waste streams were recycled and any stream containing recoverable products was recirculated.

#### Ethanol and ethyl acetate production (BEEA and EEEA)

The process flowsheet for ethanol production was modified in this scenario to produce both ethanol and ethyl acetate (Fig. 4). The hexose dominant solid stream after the pretreatment process was the input for ethanol production, while the liquid stream with pentoses was used in the acetic acid fermentation after gypsum separation. The acetic acid fermentation reactors had a retention time of 3 days, where final titer of 3% (W/W) concentration of the acetic acid was achieved. This 3% acetic acid stream was concentrated to around 30% acetic acid using series of microfiltration and nanofiltration units which had alternative pass-through of low and high rejection membranes [53]. The acetic acid rich stream was further concentrated to 35–38% (w/w) acetate acid in a multi-effect evaporator [53] and further purified to a final concentration of 98.5% (w/w) in a two-step distillation process [54, 55]. Ethyl acetate was produced through reactive distillation of the acetic acid (98.5% w/w) and ethanol (95% w/w) streams in the presence of 1% (w/w) sulfuric acid at a reactor temperature of 77 °C with a yield of 75% (W/W) ethyl acetate [56–59]. The ethyl acetate-rich stream was cooled and decanted to remove any excess moisture to obtain a 95% (w/w) ethyl acetate concentration which was further purified to 99% ethyl acetate stream after passing through molecular sieves. Pure ethyl acetate stream was cooled, stored and sold as the main product. Excess ethanol remaining after ethyl acetate production in the reactive distillation was denatured with 1% gasoline, stored and sold as a coproduct. In this scenario, four products were obtained including ethyl acetate (main product), ethanol, electricity, and gypsum.

#### Ethyl acetate production (BEA and EEA)

Ethanol from renewable sources such as corn and sugarcane is currently produced in commercially significant quantities. Therefore, this scenario was modeled on purchasing corn-based ethanol from an outside source and using it to produce ethyl acetate as described in the BEEA and EEEA scenarios above. The basic assumption for this scenario was to produce larger quantities of a high-value chemical, i.e., ethyl acetate than a commodity fuel such as ethanol while minimizing the risk associated with it and simplifying the plant operations. The raw materials cost was expected to increase due to the large volume of ethanol was purchased. In contrast to the previous scenario, hexoses and pentoses are used to produce acetic acid. Similar downstream processing procedures were followed for the purification of acetic acid [53, 56–59]. Equipments not required in this scenario such as ethanol distillation columns were removed from the process flow sheet.

#### Ethylene production (BET and EET)

This scenario focused on the production of ethylene from ethanol. The basic outline of the process flow was like the BE and EE scenarios with additional unit operations to produce ethylene from ethanol. The ethanol produced from BE and EE was heated to 400 °C where and converted to ethylene in a three reactor placed in a series configuration [60, 61]. The ethylene concentration was about 55% (w/w) after the reactors were further concentrated by adsorption and distillation [60]. Nitrogen was used as an adsorbent to remove impurities, through which 97% ethylene concentration was achieved. The overall binding efficiency was 13.05%, while 1-kg adsorbate was used for every 5-kg mass flow through the adsorption process. The ratio between breakthrough time and regeneration time was placed at 2:1 [60]. The purified ethylene stream is further concentrated in a cryogenic distillation column and stored until final sales.

#### Dodecane production (BD and ED)

Advanced jet fuels from biomass predominantly consist of C12 carbon chain hydrocarbons. Hence, dodecane was chosen as a representative chemical that was jet fuel equivalent in this scenario. The ethylene to dodecane reaction was catalyzed by nickel and diborane was used for the intermediary reactions. Dodecane formation was modeled as a two-step reaction where ethylene was first converted to trialkyl borane and in the second step, trialkyl borane was converted to 5-methylundecane, dodecane, hexane [62]. A yield of 75% pure dodecane was obtained after the reaction and was purified to 99% using distillation column and molecular sieves. The distillation columns reboiler was operated at 90 °C leaving dodecane at the bottoms, while the rest of mixtures pass through molecular sieves for further separation. Any unreacted ethylene was recycled back to the reactor. Hexane was separated out and sold as a separate by-product [62].

#### Biomass to electricity (BEL and EEL)

This scenario considers the production of electricity from lignocelluloses through the biomass to electricity pathway. This scenario was used to compare the performance of liquid fuels/chemicals production compared to other forms of energy, i.e., electricity. Here the biomass after size reduced was sent directly to the boiler for steam generation. The generated steam was expanded using a gas turbine to produce electricity. The detailed description about boiler setup in the Superpro designer is presented in scenario BE and EE. Compared with BEL, EEL had high moisture content, which resulted in less electricity production as more heat was required to burn the biomass [51, 52].

### Economic analysis

#### Assumptions and assessments

For all scenarios, the plant was considered to have an annual operation of 7920 h, while the design of the plant was based on the availability of the feedstock from the Hawaii island which was placed at 60,000 dry MT/year. The cost of the unit operations was based on the costing within the Superpro designer and previous studies [36]. A list of economic indicators used in this study is presented in Table 4. Most of the economic indicators were based on [36], while the cost of ethyl acetate and ethylene was obtained from [63]. The cost of gypsum was based on [64], while the price of dodecane (advanced biofuel) was based on the average of jet fuel prices [65]. Straight-line depreciation method was used and 10 years was assumed to the depreciation period [36]. The plant was considered to have a lifetime of 20 years, while 24 months was considered as the construction period. The startup time was assumed to be 6 months, and the salvage value of the equipment’s after its lifetime was 5% of its installed cost. The income tax was fixed at 40%, while the interest rate for the direct fixed costs was 9% per annum. The cost of utilities was based on [13, 36, 66, 67].

**Table 4:** List of important economic indicators used in this study

Type	Assumption
Annual processing capacity	60,000 dry MT/year
Biomass cost	$80/dry MT
Gypsum cost	$30/MT
Ethanol cost	$0.95/kg
Enzymes cost	$0.517/kg
Sulfuric acid cost	$35/MT
Electricity cost	$0.17/kW-h
Gasoline cost	$0.8/kg
Ethyl acetate cost	$0.96/kg
Ethylene cost	$1.39/kg
Dodecane cost	$1.48/kg
Discount rate	9%
Annual operational hours	7920/h
Depreciation method	Straight line
Salvage value	5%
Depreciation years	10 years
Life time	20 years

#### Sensitivity analysis

The sensitivity analysis reveals the robustness and susceptibility of the process and helps to understand the impact of fluctuating commodity prices on overall plant economics. Sensitivity analysis was performed for all scenarios, considering some of the principal factors which affect the economics of the process such as the biomass cost, main product cost, enzymes and the capacity of the plant. All the sensitivity analyses were performed with a ± 10%, and ± 20% variation. The ROI (return on investment) was used as the economic indicator to compare the differences between various factors in the sensitivity analysis. The variations in the biomass composition and its moisture content could also affect the ROI. Therefore, a sensitivity analysis was performed using the expected range of variations in the composition and the moisture content of biomass (Additional file 14: Table S1).

### Life cycle assessments

#### Goal, scope and system boundaries

**Fig. 11:**
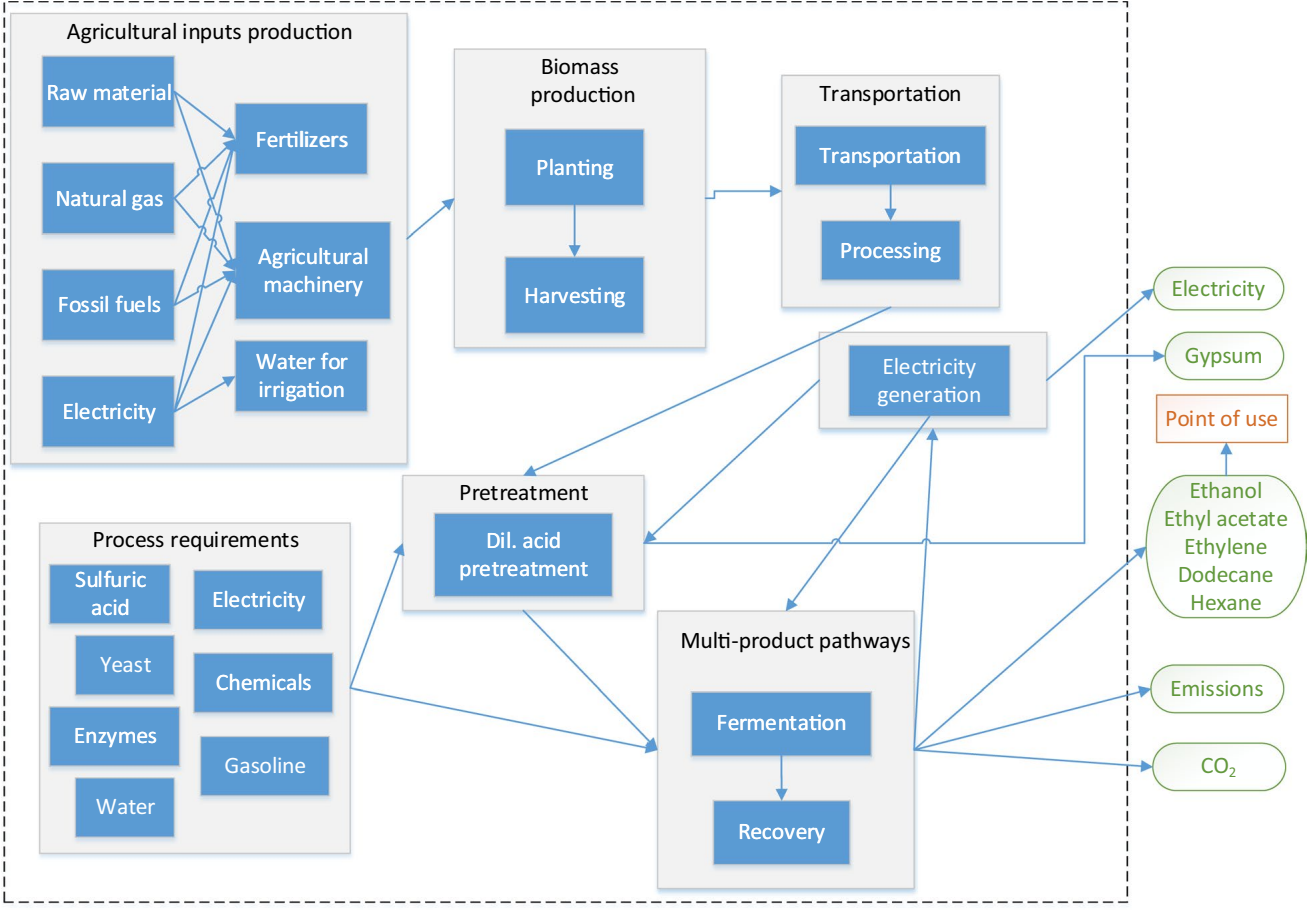
System boundary representation for the LCA study

The primary goal of this study was to develop a *well*-*to*-*pump* life cycle inventory and estimate the environmental impacts of the multi-product pathways for the two biomasses, i.e., Banagrass and Energycane. The scope of this study was to quantify and compare the environmental impacts of multi-product pathways for different biomasses in Hawaii island, which replaces conventional fossil fuel/energy sources. The functional unit used was 1 MJ for fuels/energy such as ethanol, dodecane, and electricity, whereas for chemicals such as ethyl acetate or ethylene 1 kg was considered as a functional unit. For example, in BE, 1 MJ ethanol was used as a functional unit, while for BD it was 1 MJ dodecane. Similarly, when products are produced apart from chemicals, i.e., in BEA and BEEA, 1 kg ethyl acetate was used as a functional unit. The different unit processes and its inputs were defined in the system boundaries for an LCA (Fig. 11). The agricultural inputs such as fertilizers, electricity, water for irrigation, agricultural machinery, biomass production harvesting, and transportation were included within the system boundary for the feedstock preparation. Within the production process, the chemicals used such as sulfuric acid, enzymes, yeast, gasoline, electricity, fermentation, and recovery were also included within the system boundary. In addition to the various products, byproducts such as gypsum and electricity, the emissions from the processes, i.e., CO_2_ were considered. Open LCA (Version 1.5.0) was used to run the lifecycle assessments [68], and eco-invent (V 3.1) database was used for integrating the Life cycle inventory, TRACI 2.1 was used for conducting the impact assessments.

#### Life cycle inventory

The results from the techno-economic evaluation for all the twelve scenarios were imported for LCI. The detailed description of different processes and its inventory were mentioned in “Economic viability” section (model development). The inputs and the outputs were obtained from the simulation include raw materials such as sulfuric acid, calcium hydroxide, outputs such as the amount of main product and coproduct and utilities such as the electricity produced and consumed. Any electricity exported from the plant was assumed to replace electricity from the Hawaii electricity grid. A separate process was developed for yeast production [69]. Similarly, for the biomass production, a new process consisting of crop yields, irrigation, harvest and field emissions was developed based on the data obtained from the pilot field trials conducted in Hawaii. All the other processes’ inputs and outputs were obtained from the process simulation using Superpro Designer. All the data were imported into an Ecoinvent database and integrated for conducted LCA analyses.

#### Life cycle impact assessment

The impact assessment was conducted using the Tool for the Reduction and assessment of chemical and other environmental Impact (TRACI 2.1) developed by USEPA [70]. This impact assessment method contains ten impact categories of which seven belongs to environmental impacts while the other three belongs to the human health related. The different impact categories obtained using TRACI 2.1 are as follows: acidification, ecotoxicity, eutrophication, global warming, ozone depletion, photochemical ozone formation (POF), resource depletion—fossil fuels, carcinogenics, noncarcinogenics and respiratory effects. The inputs and outputs of the different processes were attached as an electronic supplementary file along with this manuscript, which could be accessed using the eco-invent database for reproducibility and further application of the results.

## Additional files

**Additional file 1.** Superpro Model file for scenario BE.

**Additional file 2.** Superpro Model file for scenario BEEA.

**Additional file 3.** Superpro Model file for scenario BEA.

**Additional file 4.** Superpro Model file for scenario BET.

**Additional file 5.** Superpro Model file for scenario BD.

**Additional file 6.** Superpro Model file for scenario BEL.

**Additional file 7.** Superpro Model file for scenario EE.

**Additional file 8.** Superpro Model file for scenario EEEA.

**Additional file 9.** Superpro Model file for scenario EEA.

**Additional file 10.** Superpro Model file for scenario EET.

**Additional file 11.** Superpro Model file for scenario ED.

**Additional file 12.** Superpro Model file for scenario EEL.

**Additional file 13.** Historical market price comaprison of different commodities vs. crude oil.

**Additional file 14.** Sensitivity analysis of different scenarios based on lignocellulosic composition.

**Additional file 15.** Summary of LCA data used in this study.

**Additional file 16.** How to access Superpro and LCA files?

## Abbreviations

BD: Banagrass dodecane
BE: Banagrass ethanol
BEA: Banagrass ethyl acetate
BEEA: Banagrass ethanol and ethyl acetate
BEL: Banagrass electricity
BET: Banagrass ethylene
ED: Energycane dodecane
EE: Energycane ethanol
EEA: Energycane ethyl acetate
EEEA: Energycane ethanol and ethyl acetate
EEL: Energycane electricity
EET: Energycane ethylene
LCA: life cycle assessments
LCI: life cycle inventory
POF: photochemical ozone formation
ROI: return on investment
SSF: simultaneous saccharification and fermentation
TEA: techno-economic analysis
WWTP: wastewater treatment plant
