# Supplementary material for: How does technology pathway choice influence economic viability and environmental impacts of lignocellulosic biorefineries?
Source: Biotechnol Biofuels. 2017 Nov 14;10:268. doi: 10.1186/s13068-017-0959-x (PMC5686913; doi:10.1186/s13068-017-0959-x)
Supplement: Supplementary file 13 — Additional file 13. Historical market price comaprison of different commodities vs. crude oil. [file 13068_2017_959_MOESM13_ESM.docx]

Supplementary file 13: The graph here corresponds to crude oil vs different chemicals/fuels. The $/unit refers to the functional unit i.e., for ethanol and jet fuel it is $/L and ethylene is $/kg.
