# Supplementary material for: How does technology pathway choice influence economic viability and environmental impacts of lignocellulosic biorefineries?
Source: Biotechnol Biofuels. 2017 Nov 14;10:268. doi: 10.1186/s13068-017-0959-x (PMC5686913; doi:10.1186/s13068-017-0959-x)
Supplement: Supplementary file 14 — Additional file 14. Sensitivity analysis of different scenarios based on lignocellulosic composition. [file 13068_2017_959_MOESM14_ESM.docx]

|  |  | Composition | | Moisture | |
| --- | --- | --- | --- | --- | --- |
|  |  | Low | High | Low | High |
| Banagrass | Cellulose | 9.0% | 11.8% | 11.4% | 9.0% |
|  | Hemicellulose | 7.5% | 5.7% | 7.1% | 5.6% |
|  | Lignin | 4.2% | 4.3% | 5.0% | 4.0% |
|  | Extractives | 3.4% | 3.1% | 4.0% | 3.1% |
|  | Ash | 3.3% | 2.6% | 2.9% | 2.3% |
|  | Moisture | 72.7% | 72.7% | 69.7% | 76% |
| Energycane | Cellulose | 8.2% | 11.4% | 10.5% | 9.2% |
|  | Hemicellulose | 8.1% | 4.9% | 6.7% | 5.9% |
|  | Lignin | 4.2% | 3.5% | 4.0% | 3.5% |
|  | Extractives | 6.1% | 9.2% | 8.3% | 7.3% |
|  | Ash | 3.4% | 0.9% | 2.1% | 1.8% |
|  | Moisture | 70.0% | 70.0% | 68.5% | 72% |

Table 1. Composition used for the sensitivity analysis for ranges of composition and moisture content reported.

| Banagrass | Energy cane |
| --- | --- |
|  |  |
|  |  |
|  |  |
|  |  |
|  |  |

Figure 1. Sensitivity analysis based on composition and moisture content for different scenarios.
