# Supplementary material for: How does technology pathway choice influence economic viability and environmental impacts of lignocellulosic biorefineries?
Source: Biotechnol Biofuels. 2017 Nov 14;10:268. doi: 10.1186/s13068-017-0959-x (PMC5686913; doi:10.1186/s13068-017-0959-x)
Supplement: Supplementary file 16 — Additional file 16. How to access Superpro and LCA files? [file 13068_2017_959_MOESM16_ESM.docx]

# How to open Superpro files?

- Superpro Designer is a process simulation application from Intelligen Inc., USA. This study used version 8.5 (Commercial License). A license is generally required for modifying the files, while for accessing the information a demo version could be used.
- It is essential to check the version compatibility before use as the company releases latest versions.
- Superpro is widely used in several industries and universities around the globe for different applications including energy, environment, chemicals, and pharmaceutical products.
- To access the demo version, please click this link <http://www.intelligen.com/downloads.html>. Once some basic information’s are filled out, the demo version could be downloaded.
- After installation, Open the Superpro Designer application installed on your computer. To open the Superpro file, Hit File>Open>Location of the file. The Superpro file has an extension of .SPF.
- To access stream level information, right click on any stream and press simulation data.
- To run the simulation, Press F9. It should indicate a message “mass and energy balance solved successfully”.
- After that go to report>economic evaluation reports. This will provide detailed economic evaluation report. Other reports could also be produced such as cash flow analysis, itemized cost reports etc.
- Each unit operation data, can be accessed by right clicking the file and hit operation data.
- More tutorials on Superpro Designer could be obtained from <https://www.youtube.com/user/IntelligenInc>.

# How to open LCA file?

- Uncompress the 15_OpenLCA.7z to 15_openLCA.zipfiles. Rename 15_openLCA.zipfiles as 15_openLCA.zip
- Open LCA was used to assess the LCA which can be downloaded from <http://www.openlca.org/form/>.
- To run the LCA, it is essential to use ecoinvent database which acts as a spine for the analysis. The Ecoinvent data needs licence which could be available from <http://www.ecoinvent.org/database/database.html>.
- After downloading, first the ecoinvent database needs to be imported. On the left hand side ribbon, right click and hit import database. Select the location of ecoinvent database. This imports the ecoinvent database.
- After importing ecoinvent database, now download the inventory file in .JASCON format.
- This adds a separate folder to the existing database. Open the folder, and each scenario will be labelled as mentioned in the manuscript.
- Open any of the scenario, then click input/outputs tab to access the information.
- More detailed tutorials on using Open LCA could be obtained from <https://www.youtube.com/channel/UCGiahq1YZWK4pRXDVXuIi6w>
